# Critical signaling pathways governing hepatocellular carcinoma behavior; small molecule-based approaches

**DOI:** 10.1186/s12935-021-01924-w

**Published:** 2021-04-13

**Authors:** Zahra Farzaneh, Massoud Vosough, Tarun Agarwal, Maryam Farzaneh

**Affiliations:** 1 Department of Stem Cells and Developmental Biology, Cell Science Research Center, Royan Institute for Stem Cell Biology and Technology, ACECR, Tehran, Iran; 2 Department of Regenerative Medicine, Cell Science Research Center, Royan Institute for Stem Cell Biology and Technology, ACECR, Tehran, Iran; 3 Department of Biotechnology, Indian Institute of Technology Kharagpur, Kharagpur, West Bengal 721302 India; 4 Fertility, Infertility and Perinatology Research Center, Ahvaz Jundishapur University of Medical Sciences, Ahvaz, Iran

**Keywords:** Hepatocellular carcinoma, Cancer, Signaling pathways, Small molecules, Carcinoma

## Abstract

Hepatocellular carcinoma (HCC) is the second leading cause of death due to cancer. Although there are different treatment options, these strategies are not efficient in terms of restricting the tumor cell’s proliferation and metastasis. The liver tumor microenvironment contains the non-parenchymal cells with supportive or inhibitory effects on the cancerous phenotype of HCC. Several signaling pathways are dis-regulated in HCC and cause uncontrolled cell propagation, metastasis, and recurrence of liver carcinoma cells. Recent studies have established new approaches for the prevention and treatment of HCC using small molecules. Small molecules are compounds with a low molecular weight that usually inhibit the specific targets in signal transduction pathways. These components can induce cell cycle arrest, apoptosis, block metastasis, and tumor growth. Devising strategies for simultaneously targeting HCC and the non-parenchymal population of the tumor could lead to more relevant research outcomes. These strategies may open new avenues for the treatment of HCC with minimal cytotoxic effects on healthy cells. This study provides the latest findings on critical signaling pathways governing HCC behavior and using small molecules in the control of HCC both in vitro and in vivo models.

## Background

Hepatocellular carcinoma (HCC) or hepatoma is the most type of cancer in the tissues of the liver and the second leading cause of cancer-related death around the world [1, 2]. Hepatitis B/C virus and alcohol consumption are two important and independent risk factors that increase the risk of HCC [3–5]. Liver transplantation or surgical liver resection are two main options for the treatment of HCC [6, 7]. In addition to other surgical treatment options, some non-surgical methods such as chemotherapy or radiotherapy are effective treatments for HCC [8, 9]. However, these methods are not able to restrict the growth, progression, and metastasis of HCC [10]. On the other hand, these treatments cause side effects on the surrounding healthy cells [11]. Several signaling pathways are dis-regulated in HCC and lead to uncontrolled cell division and metastasis [12, 13]. Targeting specific signaling pathways that are involved in HCC phenotypes such as non-stopped cell proliferation, migration, and metastasis may control the progress of the disease [14, 15]. Recent studies have established a new approach for the prevention and treatment of HCC using small molecules [16]. Small molecules are compounds with a low molecular weight that usually inhibit the specific targets in signal transduction pathways [14, 17]. Targeting cancer-specific signaling pathways using small molecules can be novel therapeutic strategies against HCC (Table 1). Inhibition of these signaling pathways or common downstream effectors by different anti-cancer agents leads to increase apoptosis and autophagy along with a decrease in the survival, metastasis, EMT, proliferation, and colony formation of HCC cell lines and animal models [18, 19]. This study provides the latest findings on using small molecules in the control of HCC both in vitro and in vivo models.

**Table 1:** The effects of small molecules on signaling pathways related to HCC

Pathway	Small molecule		Target	Cell line	Animal model	Result	Ref.
TGF-B	Galunisertib (LY2157299)	Phase II/III in HCC NCT01722825	Receptor	SK-HEP1, HepG2, Hep3B, Huh7	–	Decrease proliferation, increase apoptosis In combination with Sorafenib, the anti-cancer effects was increased in concentration dependent manner	[42]
	PD98059	–	ERK	HepG2	7 × 10^5^ HepG2 intraperitoneal into nude mice	Inhibit proliferation, migration, invasion, and tumor growth –	[45]
Wnt	IC-2	–	TCF/β-catenin	Huh7, HepG2, HLF	Huh7 spheres to flank of NOD/SCID mice	Decrease the CSC subpopulation –	[63]
	CGP049090/ PKF115-854	-/ PMID: 23,626,717	TCF/β-catenin	Huh7, HepG2,	1 × 10^7^ HepG2 subcutaneously to Nude mice	Induce apoptosis, cell cycle arrest, inhibit tumor growth –	[191]
Hh	Cyclopamine	Phase III http://www.biomath.info/Protocols/Duke/docs/HermanSara.pdf	SMO receptor	Huh7, PLC, SM-7721,	5 × 10^6^ Mistheton Lectin-1 into the left liver of mice	Induce apoptosis, inhibit tumor growth -/-	[74, 192]
	GANT61	–	Gli	Huh7, Hep3B, HepG2	1 × 10^7^ Huh7 cells to flank of SCID mice	Induce the autophagy and apoptosis, Inhibit the HCC tumor growth Similar to Sorafenib, increase the apoptosis	[71]
	GDC-0449	Phase II https://clinicaltrials.gov/ct2/show/NCT00636610	SMO receptor	Huh7, MHCC97	5 × 10^6^ MHCC97subcutaneously to syngeneic rat	Decrease the angiogenesis Combined with Sorafenib can modulate the VEGF expression	[69]
Notch	PF-4014	Phase II https://clinicaltrials.gov/ct2/show/NCT02299635?cond=PF-03084014&draw=2&rank=1	γ-secretase	MHCC97, Huh7	1 × 10^6^ MHCC97-H or 4 × 10^5^ CSC subcutaneously to nude or SCID mice then tumor cubes were then implanted into nude mice liver lobes	Inhibited the proliferation of HCC and CSC self-renewal, decrease the tumor volume, and suppress the liver tumor metastasis PF-03084014 in combination with cisplatin or doxorubicin increase the anti-cancer effects	[80]
	GSI	Phase II https://clinicaltrials.gov/ct2/show/NCT01196416	γ-secretase	Bel7404, HepG2	–	Decrease the HCC proliferation and colony formation –	[81]
EGF	Brivanib	Phase II https://www.ncbi.nlm.nih.gov/pmc/articles/PMC5728988/	Tyrosine kinase receptor	Hep3B, HepG2, Huh7	DEN to rat	HCC apoptosis, cell cycle arrest, inhibit the liver tumor growth –	[193]
	U0126	–	Erk	HCCLM3, HepG2	–	Decrease proliferation –	[87]
	BEZ-235/ SHBM1009	PhaseII, https://clinicaltrials.gov/ct2/show/NCT01288092/-	PI3K				
HGF	PHA665752	–	c-met	MHCC97l Huh7, Hep3B	3 × 10^5^ MHCC97 subcutaneously to nude mice	Inhibit proliferation, tumor growth, and CSC, increase apoptosis –	[93]
	AMG 337	Phase I/II, https://clinicaltrials.gov/ct2/show/NCT02096666	c-met	MHCC97, HCCLM3, Hep3B, SNU, JHH5	human primary HCC tumor tissues Subcutaneously injecting nude mice	Decrease proliferation, tumor growth –	[95]
	Indo5	–	c-met	HepG2, A549, SMMC-7721 MHCC97H	2 × 10^6^ HepG2, 4 × 10^6^ MHCC 97H, 4 × 10^6^ MHCC 97 L, 2 × 10^6^ A549 cells, or 5 × 10^6^ SMMC-7721 subcutaneously to flank of SCID mouse MHCC97H subcutaneously to flank of SCID mouse then insert tumor into liver	Inhibit proliferation, migration, and metastasis Similar or better result in animal model recovery compared with Sorafenib In contrast to Sorafenib without body weight lost	[94]
VEGF	Bufalin	–	VEGFR/EGFR	SMMC-7721, PLC	5 × 10^6^ SMMC-7721 subcutaneously to flank of nude mice	Inhibit angiogenesis, HCC migration, and proliferation The anti-cancer effects of Bufalin improved in combination with Sorafenib	[194]
Stat3	Jaki	–	Jak	Huh7, Hep3B, HepG2	–	Increase apoptosis Sensitize the HCC to anti-cancer effects of Sorafenib	[112]
	C188-9	–	Stat3	PLC, HepG2, Huh7	HepPten- mice Non-alcoholic steatohepatitis (NASH)	Decrease the survival of HCC, reduce the HCC proliferation, decrease the secretion of inflammatory factors –	[113]
	S3i-201	–	Stat3	Huh7, Hep3B, HepG2	–	Induce HCC apoptosis and enhance the Sorafenib effects Increase the anti-cancer effects of Sorafenib	[112]
	UA	–	Stat3	Huh7, HepG2, SM-7721, Hep3B	1 × 10^7^ HepG2 subcutaneously into flank of nude mice	Increase the HCC apoptosis, inhibit the tumor growth –	[114]
	2-Ethoxystypandrone		Stat3	HepG2	–	Induce apoptosis and cell cycle arrest, inhibit the CSC self-renewal –	[115]
YAP/TAZ	verteporfin	–	YAP/TEAD	Huh7, MLP29	IP injection of DENA to Rats	Decrease the colony formation, survival, and tumor colony –	[125]
HIF	PT2385	Phase I, https://clinicaltrials.gov/ct2/show/NCT02293980	HIF-2a	HepG2, Sk-hep1	of 1 × 10^6^ SK-Hep1 intrahepatic injections to nude mice	Increase the efficiency of Sorafenib treatment, decrease invasion and survival Increase the anti-cancer effects of Sorafenib	[136]
Cell cycle	Dinaciclib	Phase I https://clinicaltrials.gov/ct2/show/NCT01711528?cond=Dinaciclib&draw=2&rank=2	Cdk1,2,5,9	Hep3B, HLE	1 × 10^6^ Huh7 cells or 2 × 10^6^ PLC BALB/c subcutaneously to nude mice	Decrease the colony formation, survival, induce cell cycle arrest, decrease the tumor size Similar results with Sorafenib	[143]
	Ribociclib	–	CylinD/cdk4,6	Huh7, HepG2, Hep3B, PLC	–	Decrease cell proliferation Synergist effects with Sorafenib and anti-cancer effects on Sorafenib resistance-HCC lines	[144]
Apoptosis	Tumstatin	–	Akt/mTOR	Huh7, Hep3B	5 × 10^6^ Hep3B cells subcutaneously to armpit of nude mice	Induce apoptosis, cell cycle arrest, autophagy, decrease the tumor growth, increase the apoptotic proteins –	[171]
	Brivanib	–	FGF, VEGF, P53	Huh7, HepG2, Hep3B,	Rat with DENA	Induce cell cycle arrest and apoptosis –	[82]
	Nutlin	–	MDM	Huh7, SM-7721,	–	Inhibit proliferation and survival –	[161]
	Rubone	–	miR-34a, Bcl2, cyclinD	HepG2, HuH7, Hep3B	5 × 10^6^ HepG2 to dorsal flanks of nude mice	Activate the miR34 and inhibit the TGF-B pathway and tumor growth Stronger than Sorafenib	[162]
Autophagy	Verteporfin		lysosom	HepG2, HuH7	2 × 10^6^ HepG2 to dorsal flanks of nude mice	Induce autophagy Increase the anti-cancer effects with Sorafenib	[173]
	NVP-BGT226	–	mTOR	Hep3B, HepG2, SNU475, Mahlavu	–	Induce autophagy More sensitive to Sorafenib	[174]
	Mitoxantrone	–	mTOR	HepG2, HuH7	–	Induce autophagy –	[170]
ROS	Propyl gallate	–	ROS formation	HepJ5, Hep3B, Mahlava	200 HepJ5 or Hep3B injected to yolk of zebrafish embryos	Decrease proliferation, increase apoptosis and autophagy –	[186]
	Auranofin	–	TXNRD	Hep3B	–	Increase apoptosis –	[184]

### Characterization of HCC

Hepatocytes as the most functionally liver cells have been reported to participate in HCC [20, 21].

Disruption of intracellular regulators or extracellular signals in the tumor microenvironment (TME) leads to inappropriate activation of certain signaling pathways [22–24]. Thus, aberrant molecular signaling increases levels of abnormal epigenetic modification and gene expression in the cancerous hepatocytes [25]. The outcome of these events is the loss of mature or differentiated hepatocytes (a phenomenon termed cellular dedifferentiation) [26, 27]. Under these conditions, the expression of E-cadherin (an epithelial marker) is downregulated and the cytoskeleton is reorganized [28]. The expression of *Snail*, *Twist*, and *ZEB* as the major transcription factors associated with mesenchymal cellular phenotype are up-regulated and induce an epithelial-to-mesenchymal transition (EMT) state in HCC [29]. Matrix metalloproteinases (MMPs) are also expressed at a high level in HCC and promote cellular migration and angiogenesis [30]. In HCC, the telomerase activity increases by up to 90%, checkpoints of the cell cycle are inactivated, and apoptosis is suppressed [31, 32]. All of these events cause uncontrolled cell proliferation, prolonged cell viability, and metastasis in HCC [33]. In HCC, several growth factors are released from non-parenchymal cells around the hepatocytes [14]. This event triggers cancerous phenotypes include EMT, metastasis, checkpoints aberration, uncontrolled proliferation, immortalization, and neovascularization in hepatocytes [34]. Other stimulators of hepatocyte malignancy come from microenvironmental cues such as hypoxia [35].

### Critical signaling pathways in HCC

**Fig. 1:**
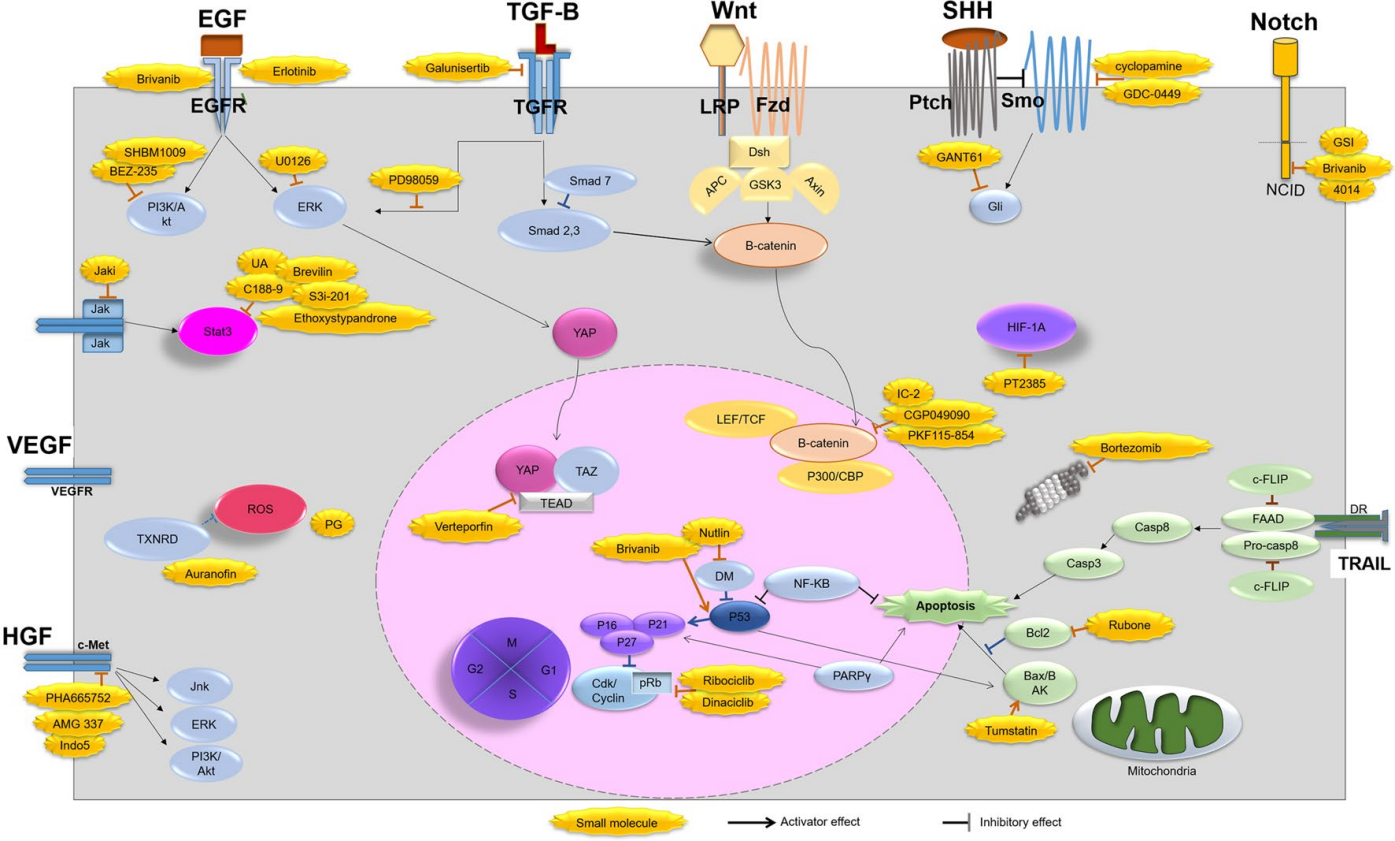
Critical signaling pathways governing HCC behavior and using small molecules in the control of HCC

Several signaling pathways, including TGF-β, Wnt/B-catenin, Hh, Notch, EGF, HGF, VEFG, JAK/STAT, Hippo, and HIF are dis-regulated in HCC and lead to uncontrolled cell division and metastasis (Fig. 1).

#### Transforming growth factor-β (TGF-β) signaling

Cancer-associated fibroblast (CAF), derived from either stromal cells or hepatocytes is the main source of TGF-β secretion in the liver tumor [36]. TGF-β binds to the heterodimer of receptors, TβRII and TβRI, phosphorylates and activates Smad2/3 that further translocate to the nucleus in association with Smad4 [37]. TGF-β upregulates the expression of *Snail*, downregulates E-cadherin in the polarized hepatocytes, and promotes EMT and metastasis [38]. The role of the TGF-β signaling pathway is also the preservation of CSC subpopulation and the promotion of HCC proliferation [39]. This pathway has been shown to induce VEGF expression in HCC and recruit endothelial cells at the tumor site [40]. TGF-β with the EGF, Wnt, and SHH pathways can promote the mesenchymal features of HCC cell lines [41]. TGF-β converts tumor-associated macrophages (TAM) to M2-like macrophages and improves proliferation, metastasis, and neoangiogenesis of HCC [38], suppresses MHC-I and II expression on HCC and modulates the immune cell defense in HCC [39]. Accumulating evidence shows that HCC cell lines represent different levels of TGF-β activity (Sk-Hep1 cells with low expression and HepG2 cells with high expression of TGF-β) [42]. Suppression of TGF-β receptors by LY2109761 or SB431542 increases E-cadherin expression, decreases migration, and invasion of HCC [43]. Recently, LY2157299 (Galunisertib) was shown to decrease both the canonical and non-canonical TGF-β pathway in HCC [42]. Galunisertib with Sorafenib has entered into the phase II clinical trial [44]. FGFR or MAPK/ERK inhibitors (such as PD98059) can also be used for inhibition of TGF-β and metastasis in HCC [45]. A combination of TGF-β inhibitor and atezolizumab (a programmed cell death ligand 1 (PD-L1) inhibitor) can overcome the immune escape of HCC [46].

#### Wnt/B-catenin signaling

In the liver tumor, HCC cells, and macrophages are emerging sources of Wnt ligand [47]. Besides, some of the environmental risk factors cause mutations in different components of the Wnt pathway, leading to overactivation of Wnt signaling in HCC [48, 49]. Binding of Wnt ligand to the Frizzled (Fzd) and low-density lipoprotein receptor-related protein (LRP) receptors causes phosphorylation of the Disheveled [50]. Activated receptors and Disheveled inhibit the destruction of complex proteins (glycogen synthase kinase 3β (GSK3β), axis inhibition protein (Axin), and adenomatous polyposis coli (APC)), thereby causing the release of β-catenin [51, 52]. Activated β-catenin further translocates to the nucleus, binds with other co-activators (like lymphoid enhancer factor (LEF)/ T-cell factor (TCF) proteins or histone acetyltransferase CREB-binding protein (CBP)/p300), and activates the transcription of several target genes [53]. These genes are involved in CSC maintenance (CD44, EpCAM), proliferation (cyclin D1, c-Myc), and EMT [54]. Leucine-rich repeat-containing G (LGR5) is a receptor related to the Wnt/B-catenin pathway and metastasis of HCC [55, 56]. High expression of LGR5 has been found in the PLC and HepG2 lines [57]. Wnt/β-catenin also regulates angiogenesis in the liver tumor [58]. TGF-β, HGF, and environmental cues (such as hypoxia condition) can activate β-catenin [59, 60]. Targeting this pathway at the receptors-ligand level or downstream effectors modulate its activation in HCC [61, 62]. It has been reported that CGP049090 and PKF115-854 can block TCF/LEF/β-catenin interactions [58]. A recent study reported that IC-2 can decrease CSC subpopulation by sphere formation assay [63]. Some of the inhibitors of β-catenin-CBP interaction can induce the differentiation of CSCs [58].

#### Hedgehog (Hh) signaling

In the liver, hepatocytes and kupffer cells are able to secrete SHH ligands after injury [64, 65]. Hepatitis B virus also activates the SHH pathway [66]. SHH interacts with Patched (Ptch) receptor and triggers Smoothened (Smo) receptor, initiates the signaling cascade, and subsequent nuclear translocation of the transcription factor, and the glioma protein (Gli) [67, 68]. SHH causes the expression of cell cycle-related genes (cyclin D, c-Myc), invasion-related genes (especially MMPs), and CSC-specific genes (like CD133) in HCC [65]. Gli enhances the expression of VEGF in HCC and tumor angiogenesis [69]. SHH can bind to the TGF-β, Wnt, or Notch pathways to promote EMT and metastasis in HCC [65]. Smo and Gli can be increased in several HCC cell lines such as Hep3B, Huh7, Sk-Hep1, and HepG2 [70]. Cyclopamine is a small molecule that inhibits SMO and GANT61 [70–72].

#### Notch signaling

Activation of the Notch pathway is regulated via the interaction of two receptors on adjacent cells, wherein one of them acts as a ligand (majorly from macrophages) and the other as a receptor, known as the Notch receptor (on hepatocytes) [73, 74]. The intracellular domain (NICD) of the Notch receptor is then cleaved by γ-secretase, which further translocates to the nucleus and binds to the DNA binding transcription factors [75]. The main target genes of the Notch pathway such as Hes1, P53, cyclin-D, and c-Myc control the expression of cancer cell proliferation, invasion, and apoptosis markers [76, 77]. However, it is notable that the Notch pathway has controversial effects on HCC [75]. This pathway crosstalks with the Wnt and SHH pathways for CSC maintenance, the PI3K and mTOR pathways for HCC proliferation, and the VEGF pathway for angiogenesis [78]. The level of the Notch pathway activity in various HCC cell lines depends on their invasion character [79]. For instance, activation of Notch signaling in an invasive MHCC97 cell line is more than the HepG2 cell line [79]. Small molecules like GSI or PF-03084014 (4014) are known to suppress γ-secretase activity [80, 81]. Brivanib is a tyrosine kinase and a Notch3 inhibitor that promotes the intracellular accumulation of P53 protein and enhances HCC apoptosis [82].

#### Epidermal growth factor (EGF) signaling

The EGF pathway can be abnormally activated in HCC via autocrine or paracrine secretion, which promotes cell proliferation and migration [83]. EGF binds to the EGF receptors and activates PI3K/Akt, MAPK/ERK, P38/MAPK, or NF-kB proteins via a series of downstream signal transduction events [84, 85]. Overexpression and overactivation of EGFR are often observed in HCC [86]. EGF pathway is involved in the recruitment of the inflammatory cells for the secretion of interleukins (IL-1, 6, 8) and tumor progression [87]. U0126 is a small molecule inhibitor of ERK; while BEZ-235 and SHBM1009 are the antagonists of PI3K [87]. EGCG can suppress the EGFR, PI3K/Akt, and MAPK/ERK pathways [88].

#### Hepatocyte growth factor (HGF) signaling

HGF was found to regulate HCC proliferation, survival, and metastasis [89, 90]. HGF binds to the c-met receptor and activates PI3K, ERK, and Jnk/Stat3 pathways [91]. c-Met inhibitors such as capmatinib and tepotinib have been assessed in liver tumor clinical trials [89, 92]. c-Met is overexpressed in the MHCC97 and HCCLM3 cell lines [89]. It has been confirmed that 3-(1H-benzimidazole-2-methylene)-5-(2-methylphenylaminosulfo)-2-indolone (Indo5), PHA665752, and AMG 337 as selective c-MET inhibitors decrease HCC proliferation, migration, and tumor growth [93–96].

#### Vascular endothelial growth factor (VEGF) signaling

In order to ensure efficient nutrient and oxygen supply in the solid tumors, the liver tumor cells secrete growth factors that promote angiogenesis [97]. Angiogenic signals can be triggered via several pathways like HGF, PDGF, FGF, and VEGF [98]. VEGF, as the main angiogenic factor, not only induces angiogenesis, but also interacts with RTK in an autocrine manner, and activates PI3K/Akt pathway in HCC [99, 100]. Sorafenib is known to inhibit the VEGF, PDGF, and FGF pathways, thereby suppressing neovasculogeneis in HCC [101, 102]. LY2109761 (TGF-β inhibitor) can suppress VEGF secretion and neovascularization in HCC [103].

### Targeting common downstream proteins in HCC

Several growth factors or environmental signaling pathways can activate the common targets in HCC [104, 105]. Signal transducer and activator of transcription 3 (Stat3), Hippo, and HIF are the main downstream proteins that are activated in HCC [106]. Inhibition of these proteins can suppress or weaken the activated signal pathway, thereby modulating the tumorigenicity of HCC [107, 108].

#### Janus kinases (Jak)/Stat3 signaling

The Jak/Stat3 pathway can be stimulated by inflammatory cytokines (such as interleukins, tumor necrosis factor (TNF), HGF, TGF-β, and EGF) [109, 110]. Stat3 as a transcription factor can promote HCC proliferation, metastasis, tumor survival, and angiogenesis [111]. The Jak inhibitors such as Jaki and S3i-201, or Stat3 inhibitor-related small molecules such as C188-9, ursolic acid (UA), and 2-Ethoxystypandrone can induce apoptosis, cell cycle arrest, and block CSC self-renewal in HCC [112–115].

#### Hippo signaling

Several growth factors such as Wnt, Notch, EGF, and SHH can activate the YAP (Yes-associated protein) pathway [116, 117]. Activated YAP translocates to the nucleus and interacts with a transcriptional coactivator, PDZ-binding motif (TAZ), and transcriptional enhanced associate domain (TEAD) to promote proliferation, metastasis, and inhibition of apoptosis and autophagy in HCC [118]. YAP or TAZ are highly expressed in HCC cell lines such as HLF and HepG2 and also primary liver tumor samples [119, 120]. Hippo protein activates several kinases and negatively regulates the expression of oncoprotein YAP [121]. Inhibition of YAP/TAZ/TEAD transcriptional activity is often used for anti-cancer treatment [122–124]. Verteporfin is a small molecule that inhibits YAP/TEAD complex interaction [125].

#### Hypoxia signaling

It has been confirmed that HCC cells rapidly use environmental oxygen [126]. In the center of the liver tumor, hypoxic conditions activate major transcription factors and inducing factors such as HIF-1A, HIF-2A [127]. HIF induces the expression of TGF-β and Snail and enhances EMT in tumor cells [126]. HIF via MMP expression helps in ECM remodeling and tumor cell invasion [128]. It also increases c-Myc expression, HCC proliferation, and escape of HCC from the immune destruction [129]. HIF also inhibits P53 (a tumor suppressor gene), enhances the activity of anti-apoptotic proteins (like Bcl-2, caspases), and prevents HCC apoptosis [126]. HIF-1A promotes CSCs maintenance in liver tumors [130]. HSP90, a general oncogene protein, stabilizes HIF-1A and positively modulates the survival, growth, and metastasis of the tumor cells [131]. Under hypoxia conditions, the cells transition from aerobic to anaerobic metabolism [132]. HIF-1A promotes glycolysis metabolism and increases lactate production in HCC [133]. The components of this pathway also interact with other pathways to promote tumorigenicity [134]. HIF-1A impacts on downstream signal transduction and increases VEGF expression and angiogenesis in HCC [98]. HIF-1A also stimulates TGF-β interaction with its receptors, enhances HCC survival, and proliferation [128]. Hypoxia activates the expression of Notch downstream genes and recruits HIF-1A for HCC metastasis [130]. Recent studies have suggested that hypoxia can regulate the Hh pathway [130]. The Wnt pathway also increases the expression of HIF-1A in HCC [130]. YAP interacts and stabilizes HIF-1A in HCC [135]. HIF-1A regulates the metabolism of HCC, increases the expression of glycolysis enzymes and glucose uptake receptors for adaptation to the hypoxic condition [130]. Besides, HIF-1A changes the activity of the macrophages and hepatic stellate cells (HSC) to promote HCC survival, growth, and angiogenesis [130]. PT2385 as a small molecule can suppress HIF-activated proteins such as Stat3, VEGF, PDGF, and ERK [136].

#### Cell division signaling

Uncontrolled cell cycle program and telomerase activity in the hepatocytes increase carcinogenesis [137]. The cell cycle is regulated by cyclin-dependent kinases (CDK)/Cyclin complex at different stages [138]. Downstream of signaling pathways such as EGF, TGF-β, TNF, and IL6 can stimulate CDK/CyclinD complex and phosphorylated retinoblastoma (pRb) to promote HCC proliferation [139]. P53, an anti-proliferation protein, activates P16, P21, and P27 tumor suppressor proteins at the G1 phase, thereby hindering the pRb and CDK/Cyclin proteins [140]. Notably, P21 via inhibition of procaspase 3 has contradictory effects in cancers [141]. Normal hepatocytes have a cell cycle arrest in the G0 phase; however, in the case of HCC, P21, and P27 are usually degraded [142]. Mutations of β-catenin or P53 lead to sustain expression of c-Myc, misregulation of PI3K and ERK pathways, and uncontrolled cell cycle progression in HCC [138].

In this regard, Dinaciclib and Ribociclib are CDK/pRb inhibitors that upregulate P53 to control HCC proliferation [143, 144].

#### Apoptosis signaling

Targeted activation of the apoptosis pathway in cancer cells is another crucial way in cancer therapy [145, 146]. In normal cells, apoptosis may initiate via the extrinsic (owing to the attachment of external ligands to the receptors) or intrinsic (owing to mitochondrial factors) pathways [147]. Cellular FlICE/caspase-8-inhibitory protein (cFLIP) and Bcl-2 are negative regulators of the apoptosis pathway, while PPARγ acts as an apoptosis inducer [148, 149].

The extrinsic pathway is activated when immune cells secrete TNF-related apoptosis-inducing ligand (TRAIL) that binds to death receptors (DR) on the cell surface [150]. This cascade causes the recruitment of a complex of FAAD-procaspase 8 (DISC complex), and subsequent activation of caspase 8 (an endonuclease and protease), leading to apoptosis [148]. The proteasome complex causes the degradation of tumor suppressor proteins and activation of NF-kB and c-FLIP, thereby promoting the survival and proliferation of HCC [148]. Additionally, NF-kB regulates MMP9 expression and HCC metastasis [151]. On the other hand, in the intrinsic apoptosis pathway, DNA damage in the cells activates P53 protein, triggers the activation of Bax, and mitochondria-mediated caspase activity [152]. P53 is crucial for cell cycle arrest, cell senescence, and cell autophagy [153, 154]. In HCC, mutations or deletion in the P53 gene or increase of its inhibitors such as a ubiquitin ligase DM2 (Double Minute 2) obligate the apoptosis pathway [155]. Snail inhibits the TRAIL pathway and P53 in cancer cells [156] HCC cell lines express P53 at different levels. For instance, Hep3B, HepG2, and Huh7 have no, normal, and high levels of P53, respectively [157]. PPARγ also positively modulates the components of these pathways and inhibits HCC survival [158]. The strategies that upregulate TRAIL receptors or ligands (via recombinant protein or agonist receptor antibodies) were shown to cause selective apoptosis in HCC cell lines [159, 160]. Co-treatment of HCC cell lines with recombinant TRAIL and Bortezomib (as proteasome inhibitors) increased the apoptosis induction in the Huh7 cells, compared to the primary hepatocytes [159]. Nutlin, an inhibitor of DM, was reported to stabilize P53 and decrease Bcl-2 expression [161]. Rubone can downregulate the expression of Notch, cyclin D1, Bcl-2, while increase P53 level in HCC [162].

#### Autophagy signaling

Autophagy, a type II cell death, is lysosome-dependent and initiated by surrounding the intracellular organelle with a double membrane (autophagosome) and self-degradation of cells [163]. ATG7, LC3, and beclin are the major proteins involved in this process [164]. Depends on the stage of cancer, autophagy either negatively or positively regulates cancer progression [165, 166]. In HCC late stages, autophagy promotes survival, metastasis, and EMT via activation of the TGFβ pathway, P53 degradation, and chemotherapy resistance of HCC [167]. Inhibitors of main signaling pathways such as PI3K/Akt, MAPK/ERK, and JAK/Stat3 can induce autophagy and cell death in HCC [168, 169]. Small molecules like rapamycin, Mitoxantrone (PI3K/mTOR inhibitors), and Erlotinib/Cetuximab (EGFR inhibitors) are thought to activate cellular autophagy and apoptosis in various HCC cell lines [167, 170]. Tumstatin was previously shown to increase the expression of Bax, Fas, and Fasl to induce apoptosis and autophagy in HCC [171]. However, some studies have found that the suppression of autophagy via 3-MA leads to inhibit HCC growth [167, 172]. Verteporfin, mitoxantrone, and NVP-BGT226 are small molecules that trigger autophagy in HCC [170, 173, 174].

#### Oxidative stress signaling

Both the intrinsic and extrinsic apoptotic pathways affect the mitochondrial respiratory chain and cause the generation of reactive oxygen species (ROS) in the cells [152, 175]. In cancer cells, ROS may play as a double-edged sword in the induction or suppression of tumor growth in a concentration-dependent manner [176]. A low level of ROS is normal in all the cell types, while its moderate level leads to promote cancer development [176]. ROS, via activation of the TGF-β pathway along with an increase in MMP expression, causes EMT, metastasis, and invasion of cancer cells [176]. ROS can stimulate VEGF or the hypoxia pathway to promote angiogenesis in HCC [177, 178] and mediates cell cycle activation and CSC maintenance in cancer [179]. ROS-mediated signaling events mediate chemoresistance to the cancer cells [180]. Though, excessive ROS can disrupt the proteins in mitochondria and promote the DNA mutations, causing the release of pro-apoptotic factors into the cytoplasm of the cancer cells [178]. Accordingly, agents that restore the intracellular REDOX balance or elevate the ROS content cannot be useful in cancer treatment [181, 182]. In this regard, vitamin C as a natural antioxidant can increase ROS production in HCC and stimulate apoptosis, cell cycle arrest, and suppress CSC self-renewal [183]. Auranofin, a thioredoxin reductase (TXNRD) inhibitor, increases ROS in HCC and suppresses both the extrinsic and intrinsic apoptotic pathways [184]. Morin, a flavonoid from *Ficus carica*, in combination with Auranofin caused apoptosis in HCC [185]. Propyl gallate (PG), a synthetic antioxidant, activates superoxidase and ROS formation in HCC, thereby causing autophagy and apoptosis [186]. N-acetylcysteine (NAC) acts as a potent ROS inhibitor [187]. ART, a YAP inhibitor, promotes ROS formation in HCC [188].

## Conclusion and perspective

Several important signaling pathways such as TGF-β, Wnt, SHH, Notch, and RTK are misregulated in HCC, compared to the normal hepatocytes. These pathways initiate differential networks that consequently result in HCC cell cycle promotion, EMT, metastasis, vasculogenesis, and anti-apoptotic mechanisms. Suppression of these pathways with small molecules, herbal drugs, and miRNA stimulates cell cycle arrest, apoptosis, and inhibits the invasion of HCC [189, 190]. Simultaneously targeting different signaling pathways or common downstream proteins would facilitate control over malignant HCC. Induction of differentiation in transformed mesenchymal HCC to the epithelial state would also help in regulating the tumorigenesis of HCC. Smart delivery of anti-cancer agents to the liver tumor could facilitate the targeted therapy in this solid tumor.

## Data Availability

The datasets used and/or analyzed during the current study are available from the corresponding author on reasonable request.

## Abbreviations

APC: Adenomatous polyposis coli
CAF: Cancer-associated fibroblast
CBP: CREB-binding protein
CDK: Cyclin-dependent kinases
Cflip: Cellular FlICE/caspase-8-inhibitory protein
DM2: Double Minute 2
DR: Death receptors
HSC: Hepatic stellate cells
ROS: Reactive oxygen species
EGF: Epidermal growth factor
EMT: Epithelial-to-mesenchymal transition
Fzd: Frizzled
Gli: Glioma protein
GSK3β: Glycogen synthase kinase 3β
HGF: Hepatocyte growth factor
HCC: Hepatocellular carcinoma
Hh: Hedgehog
Indo5: 3-(1H-benzimidazole-2-methylene)-5 (2methylphenylaminosulfo)-2-indolone
Jak: Janus kinases
LEF: Lymphoid enhancer factor
LGR5: Leucine-rich repeat-containing G
LRP: Lipoprotein receptor-related protein
MMPs: Matrix metalloproteinases
PD-L1: Programmed cell death ligand 1
PRb: Phosphorylated retinoblastoma
Ptch: Patched
Smo: Smoothened
TAM: Tumor-associated macrophages
TCF: T-cell factor
TEAD: Transcriptional enhanced associate domain
TGF-β: Transforming growth factor-β
TME: Tumor microenvironment
TRAIL: TNF-related apoptosis-inducing ligand
TXNRD: Thioredoxin reductase
UA: Ursolic acid
VEGF: Vascular endothelial growth factor
YAP: Yes-associated protein
